# Acute dissection of a syphilitic saccular aneurysm of the ascending aorta and arch in a hypertensive patient - a rare phenomenon

**DOI:** 10.4322/acr.2024.475

**Published:** 2024-02-26

**Authors:** Hubert Daisley, Dennecia George, Johann Daisley

**Affiliations:** 1 Scarborough General Hospital, Scarborough, Trinidad and Tobago

**Keywords:** Syphilis, Cardiovascular, Hypertension, Aneurysm, Aortic Dissection, Hypertrophy, Left ventricular

## Abstract

We report the case of a 77-year-old male who suffered from hypertension and died suddenly. At autopsy, he was found to have hypertensive cardiomegaly and a dissecting syphilitic saccular aneurysm of the ascending aorta and arch with tamponade. Chronic aortic regurgitation, which is often seen in syphilitic aortitis, produces an additive effect to the concentric left ventricular hypertrophy seen in hypertension.

## INTRODUCTION

Acute aortic dissection with cardiac tamponade is a common autopsy finding in patients who suffered from hypertension. Based on the Stanford system, type A intimal tear occurs in the intima of the ascending aorta, or a type B intimal tear occurs distal to the origin of the left subclavian artery. Entry of blood occurs between the intima and the media, and dissection and separation of the aortic wall layers occur. Dissection with perforation of the adventitia can occur at the root of the aorta with resultant hemopericardium and cardiac tamponade. This catastrophic event is often fatal.^1-3^ While aortic dissection often occurs in hypertension, it is also seen in genetic conditions such as Marfan syndrome, pregnancy, aortic instrumentation, and inflammatory or infectious diseases that cause vasculitis, such as syphilis.^3^

Aortic dissection in syphilis is uncommon,^4-7^ and acute aortic dissection occurring in patients who simultaneously have hypertension and syphilitic saccular aneurysm is rare and has seldom been reported.^4,6,8^

We report a case of acute aortic syphilitic saccular aneurysm dissection in a 77-year-old male who suffered from hypertension and died suddenly. At autopsy, in addition to his hypertensive heart, he was also found to have an acute dissection of a syphilitic saccular aneurysm of the ascending aorta and arch, with hemopericardium. An additional finding was a fracture of his cervical vertebrae between C4 and C5 from falling during the catastrophic event.

## CASE REPORT

A 77-year-old male diagnosed with hypertension was treated with amlodipine. On the day of his demise, he suffered no chest pain, shortness of breath or tiredness. He was found dead in the early hours of the morning, lying face down on his bedroom floor.

## AUTOPSY FINDINGS

The body was that of a six-feet-long elderly male with an asthenic build. There were three facial contusions: two contusions in the region of the right supraorbital region, each 0.015 m long, and one over the nasal bone 0.01m long. There was cyanosis at the nail beds. There was a transverse fracture between the cervical vertebrae, anteriorly between C4 and C5. There were no skull fractures, nor were there brain hemorrhages or infarctions. There was cerebral edema with the brain weighing 1.4kg (RR 1.44 +/-0.03 kg).

**Figure 1:**
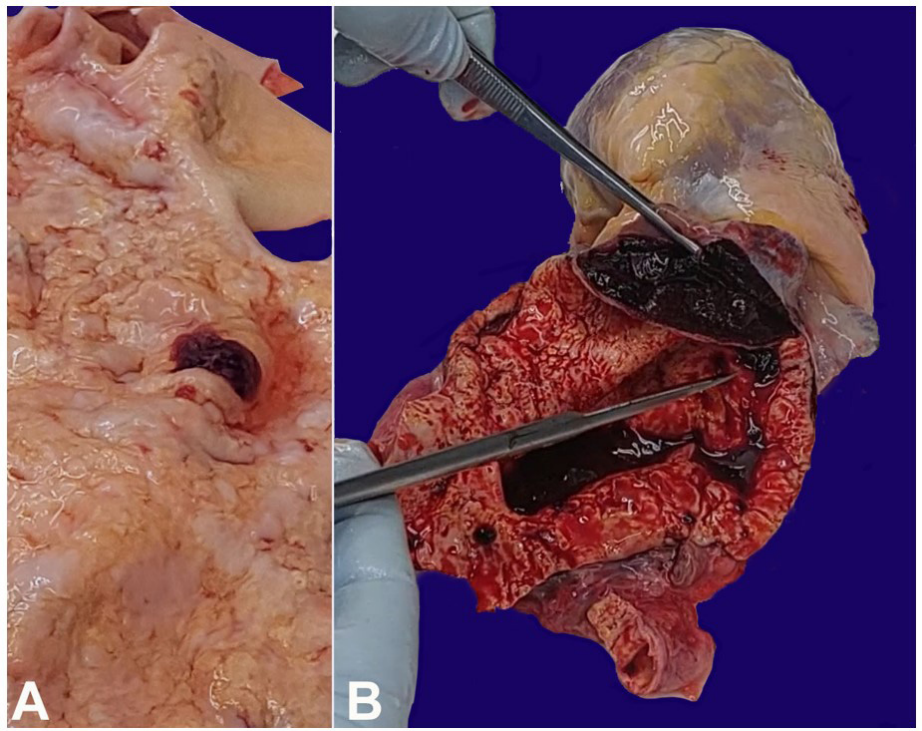
**A** - shows the intima of the saccular aneurysm with the intimal tear (hemorrhagic area), and the wrinkling of the intima (tree barking phenomenon); **B** - shows a saccular aneurysm with dissection between the intima and the media, and deposits of atheroma with wrinkling of the intima giving rise to the tree bark-like appearance.

There was a 0.14x0.1 m^2^ saccular aneurysm of the ascending aorta and aortic arch, with 0.4 kg of blood and clot in the pericardial sac. There was a 0.02m intimal tear within the saccular aneurysm (Stanford A) (Figure 1A) and dissection of the saccular aneurysm with a 0.015 m tear in the ascending aorta adventitia just above the aortic ring. The dissection was confined to the saccular aneurysm (Figure 1B). There were diffuse wrinkling and atheroma deposits within the intima of the saccular thoracic aneurysm and thoracic aorta viz tree barking (Figure 1B). There were no other aneurysms of the aorta or its branches.

The coronary ostia were patent but contained circumferential atheromatous deposits. The left anterior descending coronary artery was 20% stenosed with atheroma. The left circumflex and right coronary arteries were patent. There was concentric left ventricular hypertrophy of the myocardium with the left ventricular thickness 0.17m (>0.14m is hypertrophy). There was no myocardial injury, past or present. The mitral valve circumference measured 0.09 m (RR 0.078+/-0.007m), and the aortic valve was 0.08 m (RR 0.053+/-0.004m). There was no valvular vegetation. The heart weighed 0.56kg (RR 0.125- 0.250 kg).

The right lung weighed 0.66 kg (0.455 kg is the upper limit of normal), and the left weighed 0.6 kg (0.415 kg is the upper limit of normal). There was chronic obstructive pulmonary disease with congestion. The liver weighed 1.56 kg (1.52 kg is the upper limit of normal) and showed chronic passive venous congestion. The kidneys weighed 0.16 kg (RR 0.098-0.112 kg) and 0.12kg (RR 0.09-0.110 kg) on the left and right, respectively, and benign nephrosclerosis was found. The spleen was firm, congested and weighed 0.16 kg (RR 0.15-0.2 kg).

Reference weights for organs stated in brackets were taken from Autopsy Pathology: A Manual and Atlas (2^nd^ edition) by Finkbeiner et al.^9^

## HISTOLOGY

### Saccular aortic aneurysm:

**Figure 2:**
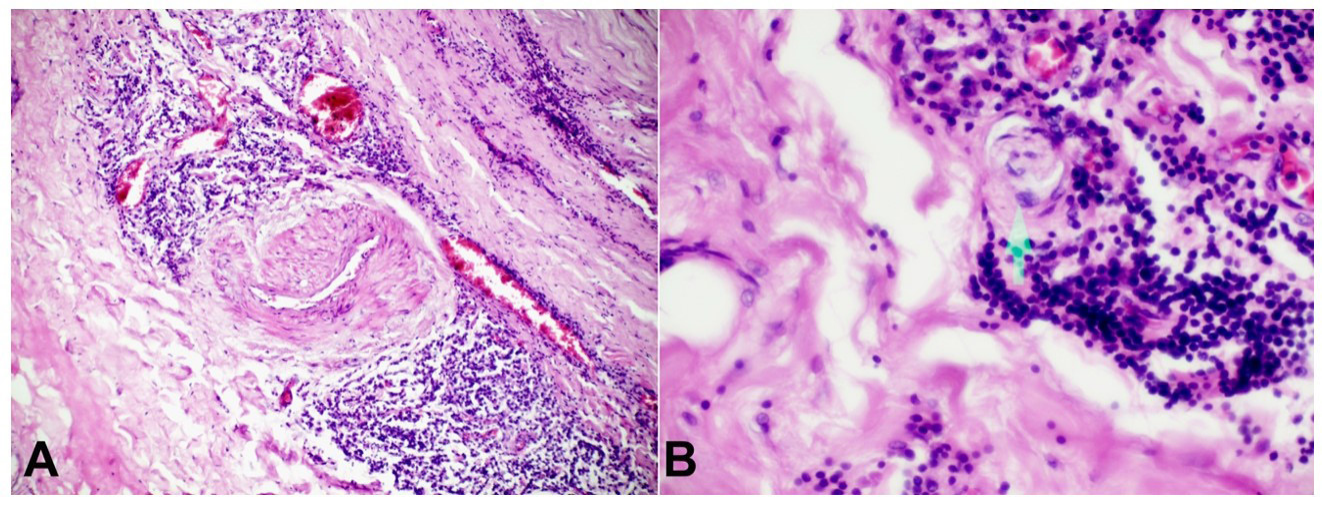
**A -** shows chronic inflammation mainly of lymphocytes and plasma cells around the vasa vasorum, extending to numerous capillaries. There is also fibrosis of the media (H&E 10x40); B - shows Endarteritis Obliterans (H&E 10 X 40).

Sections of the saccular aneurysm showed diffuse lymphoplasmacytic cells infiltrating around the vasa vasorum, interrupted by fibrosis of the media and adventitia. There was also endarteritis obliterans. These findings are presumptive of syphilitic aortitis. (Figure 2A and 2B).

## DISCUSSION

The case in discussion is one of a 77-year-old hypertensive male who had sudden death from syphilitic aortitis, with a dissecting saccular aneurysm of his ascending aorta and arch, with cardiac tamponade. He fell during this catastrophic event, sustaining facial injuries and fracturing the cervical vertebrae between C4 and C5. He had cardiomegaly with concentric left ventricular hypertrophy. Although concentric left ventricular hypertrophy is indicative of hypertensive cardiac disease, chronic aortic regurgitation from the syphilitic aortitis can also augment concentric left ventricular hypertrophy.^10-12^ This patient suffered from hypertension and was treated for many years with antihypertensive medication. According to Kontos et al.,^12^ patients with mild to moderate aortic regurgitation who suffer from hypertension have larger left ventricular muscle mass. They are more prone to the risk factors of hypertension viz aortic dissection.

Acute aortic dissection is a feature of both hypertension and syphilitic aortitis.^5,6,13-16^ Concurrent hypertensive cardiac disease and syphilitic saccular aneurysm of the aorta with acute dissection and hemopericardium in patients is a rare finding that has seldom been reported, and pathologists should be aware of this combination and their shared cardiac pathology.^4,6,8^ When aortic dissection occurs in such a patient, it is impossible to tell whether it was hypertension or the syphilitic aortitis or the combination that caused the acute aortic dissection.

Other predisposing factors for acute aortic dissection of the ascending aorta are pregnancy, connective tissue diseases, and instrumentation of the aortic valve.^15-20^

Although sexually transmitted diseases are prevalent, in recent times, emphasis has been placed on the detection of HIV, with less attention being placed on syphilis. As a result, primary syphilis may go undetected^21,22^ and untreated, with progression to secondary and tertiary syphilis, with its devastating cardiovascular consequences.^23^

## CONCLUSION

Healthcare workers must pay attention to the diagnosis of syphilis to avert its devastating long-term consequences.

Chronic aortic regurgitation from syphilitic aortitis can augment concentric cardiac left ventricular hypertrophy in hypertension.

Coexistence of aortic dissection in patients who suffer from hypertension and syphilitic aortitis is uncommon.
